# Epidemiological characteristics of human-to-human transmission of severe fever with thrombocytopenia syndrome in China from 1996 to 2023

**DOI:** 10.1371/journal.pntd.0013283

**Published:** 2025-07-24

**Authors:** Li Yuan, Tingting Tian, Aqian Li, Shanshan Du, Shiwen Wang, Dexin Li, Xiaoxia Huang, Jiandong Li

**Affiliations:** 1 National Institute for Viral Disease Control and Prevention, Chinese Center for Disease Control and Prevention, Beijing, People’s Republic of China; 2 National Key Laboratory of Intelligent Tracking and Forecasting for Infectious Diseases, Beijing, People’s Republic of China; Universite de Montreal, CANADA

## Abstract

**Background:**

Severe fever with thrombocytopenia syndrome (SFTS) is an emerging viral disease transmitted mainly through the bite of *Haemaphysalis longicornis*, and can cause clusters through contact transmission, and its incidence shows a rising and spreading trend in China. This study aimed to analyze the occurrence process of human-to-human transmission clusters and provide evidence for effective implementation of interventions.

**Methods:**

Data about SFTS human-to-human transmission clusters were extracted from 42 published articles. R 4.4.1 and Microsoft Excel software were used to process and analyze the epidemiological and clinical data extracted.

**Results:**

37 clusters occurring from 1996 to 2023 were retrieved, of which 2 (5.41%) involved third-generation transmission, 7 (18.92%) involved 16 asymptomatic infections, and 9 (24.32%) involved 17 medical personnel. There were 37 index cases with a case fatality rate of 97.30%, 135 secondary cases with a case fatality rate of 12.40%, and an overall of 31.33%. The first treatment of the index case was mainly in primary medical institutions (24, 64.86%) and the most common symptoms were fever, fatigue and gastrointestinal symptoms. The index cases were distributed from March to October each year, the peak was from April to July, and the incubation period was 5–21 days, mostly in middle-aged and elderly farmers. Clusters were mainly distributed in Jiangsu Province (9 clusters), followed by Henan, Shandong and Zhejiang Provinces (6 clusters each). The clusters occurred mostly in the progress of care (72.97%), funeral (64.86%) and treatment of patients (24.32%), involving relatives (75.76%), medical workers (12.12%), villagers (9.85%) and morticians (2.27%). Almost all clusters were spread by contact with patients’ blood and bloody secretions (97.30%).

**Conclusions:**

SFTS human-to-human transmission clusters sometimes occur in China, with a high case fatality rate. It is necessary to strengthen public health education, and improve the early diagnosis and treatment ability of medical workers, to avoid nosocomial infection or family (community) transmission.

## Author summary

This study explored the epidemiological and clinical characteristics of 37 SFTS human-to-human transmission clusters that occurred in China from 1996 to 2023. We found that the clusters occurred from March to November and were mainly concentrated in rural areas in central, eastern and northeastern China, and the affected population was mainly middle-aged and elderly farmers. The number of cases involved in a single cluster ranged from 2 to 13, with an average of 5 cases. Because the early symptoms were atypical, most of the index cases were diagnosed after multiple medical visits. The case fatality of the index cases (97.30%) was extremely high. Secondary cases were mostly infected by contact with the blood and bloody secretions of patients, and mostly occurred among family members, medical workers, villagers, and morticians who were involved in nursing care (72.97%), funeral and mortuary (64.86%), and medical treatment (24.32%). If early diagnosis, treatment and management can be achieved, while raising public awareness of SFTS and taking personal precautions when caring for cases and contacting the remains of SFTS cases, the occurrence of human-to-human transmission clusters can be avoided.

## Introduction

Severe fever with thrombocytopenia syndrome (SFTS) is a zoonotic disease caused by *Dabie bandavirus* (also known as SFTS virus) firstly identified in China with a high case fatality rate (30%) [1]. Clinical manifestation of SFTS is often complex and atypical, mainly characterized by fever, thrombocytopenia, leukocytopenia, fatigue, and gastrointestinal symptoms [1]. SFTS virus belongs to genus *Bandavirus* of the family Phenuiviridae, mainly transmitted by *Haemaphysalis longicornis* ticks [2,3], and also can cause human-to-human transmission through contact with SFTS patient’s blood, bloody secretions, and so on [4–6]. Due to its high fatality and human-to-human transmission, SFTS has been considered as a challenge to public health.

In 2010, China issued guidelines for SFTS diagnosis and reporting across all region and SFTS case is required to be reported to Chinese Disease Prevention and Control Information System, which is an infectious disease surveillance network operating from the county to provincial levels in China [7]. From 2010 to 2021, a total of 18,973 SFTS cases have been reported in 27 provinces in China, about 99% distributed in Shandong, Anhui, Henan, Hubei, Liaoning, Zhejiang, and Jiangsu 7 provinces. These cases were predominantly middle-aged and elderly farmers [8,9]. SFTS has been expanding in the region and the number of cases is rapidly increasing in recent years. Besides China, autochthonous SFTS cases have been reported in several Asian countries, including Japan, South Korea, Vietnam, Myanmar and Thailand [10–14]. In Africa, antibodies to SFTS virus were detected in the general population of Kenya [15].

The number of SFTS cases in China is far more than that of Japan and South Korea [16]. In China, SFTS cases are mainly distributed in seven provinces mentioned above. It is highly sporadic and can occur throughout the year, but mainly from April to October, accounting for 97% of the total number of cases, peaked at May to July [8]. *Haemaphysalis longicornis* ticks, the primary vector of SFTS virus, have a broad host range including livestock such as cattle, sheep, and dogs, as well as various wild animals [17]. They are widely distributed across China, particularly in North China and Central China [18]. Studies [19,20] have shown that ticks usually prefer to inhabit in warm and humid environments with abundant vegetation and are mostly active in spring and autumn. People living, working, or traveling in areas such as hills, mountains, and forests have a higher exposure chance to ticks and are at a higher risk of SFTS virus infection.

Although SFTS is mainly sporadic, clusters caused by human-to-human transmission occurred occasionally in some regions in China, which aroused public health concerns. In order to further clarify the occurrence process and dynamic characteristics of SFTS human-to-human transmission clusters in China, formulate targeted preventive and control measures, and avoid the occurrence of such preventable public health events, this study systematically analyzed clusters of SFTS human-to-human transmission in China that were publicly published between January 2010 and September 2024.

## Methods

### Case definition

SFTS cases were classified as suspected cases and lab-confirmed cases in China, based on their epidemiological information, clinical manifestations and laboratory test results [7]. The case definitions of them have been detailed described in our previous studies [21]. In this study, “clusters of SFTS human-to-human transmission” is defined as a transmission occurred from an index SFTS case to at least one close contacts who was lab-confirmed SFTS cases, within 21 days.

### Study objects

Information related to clusters of SFTS human-to-human transmission occurring in China from domestic and foreign published literature was obtained. The period of literature search was from January 1, 2010 to September 30, 2024, including both Chinese and English languages.

### Search strategy

Literature about clusters of SFTS human-to-human transmission was searched in four databases including China National Knowledge Infrastructure (CNKI), Wanfang Data, Web of Science and PubMed. Search terms “severe fever with thrombocytopenia syndrome & person-to-person transmission” and “severe fever with thrombocytopenia syndrome & human-to-human transmission” were used to search literature written both in English and Chinese. Furthermore, considering that human-to-human transmission is often directly translated into “cluster” used in Chinese articles, in order to avoid missing relevant articles search terms “severe fever with thrombocytopenia syndrome & family cluster” and “severe fever with thrombocytopenia syndrome & cluster epidemic” were also used in searching literature in Chinese. In addition, the references of the included literature were also retrospectively checked. Literature unrelated to clusters of SFTS human-to-human transmission, review literature, and literature with scarce information or incomplete data were excluded.

### Data extraction

According to the above search strategy, targeted literature was searched and screened. Firstly, duplications were removed. Secondly, literature unrelated to SFTS human-to-human transmission and review literature were excluded by reading the title and abstract. Thirdly, literature without relevant detailed information was excluded by reading the full text. After that, the remaining literature was included in this study. The data including authors, publication time, study region, basic information of SFTS cases, consultation information, clinical manifestations, epidemiological history, and information about secondary cases were extracted for the following descriptions and statistical analysis. Two researchers independently conducted literature screening and data extraction. For controversial literature or data, the decision of inclusion was made through internal discussion or consultation with experts.

### Data analysis

Data was analyzed using R 4.4.1 and Microsoft Excel software. Descriptive epidemiological methods were used to analyze the epidemiological and clinical characteristics of clusters. Qualitative data was expressed by frequency and quantitative data was described by mean±standard deviation. To determine the difference between the index and secondary cases, Pearson χ^2^ test, Independent Sample T test and Wilcoxon W test were used to compare qualitative variables, normal and abnormal distribution continuous variables, respectively. In statistics, *P* < 0.05 indicates a significant difference.

## Results

### Inclusion of literature

**Fig 1:**
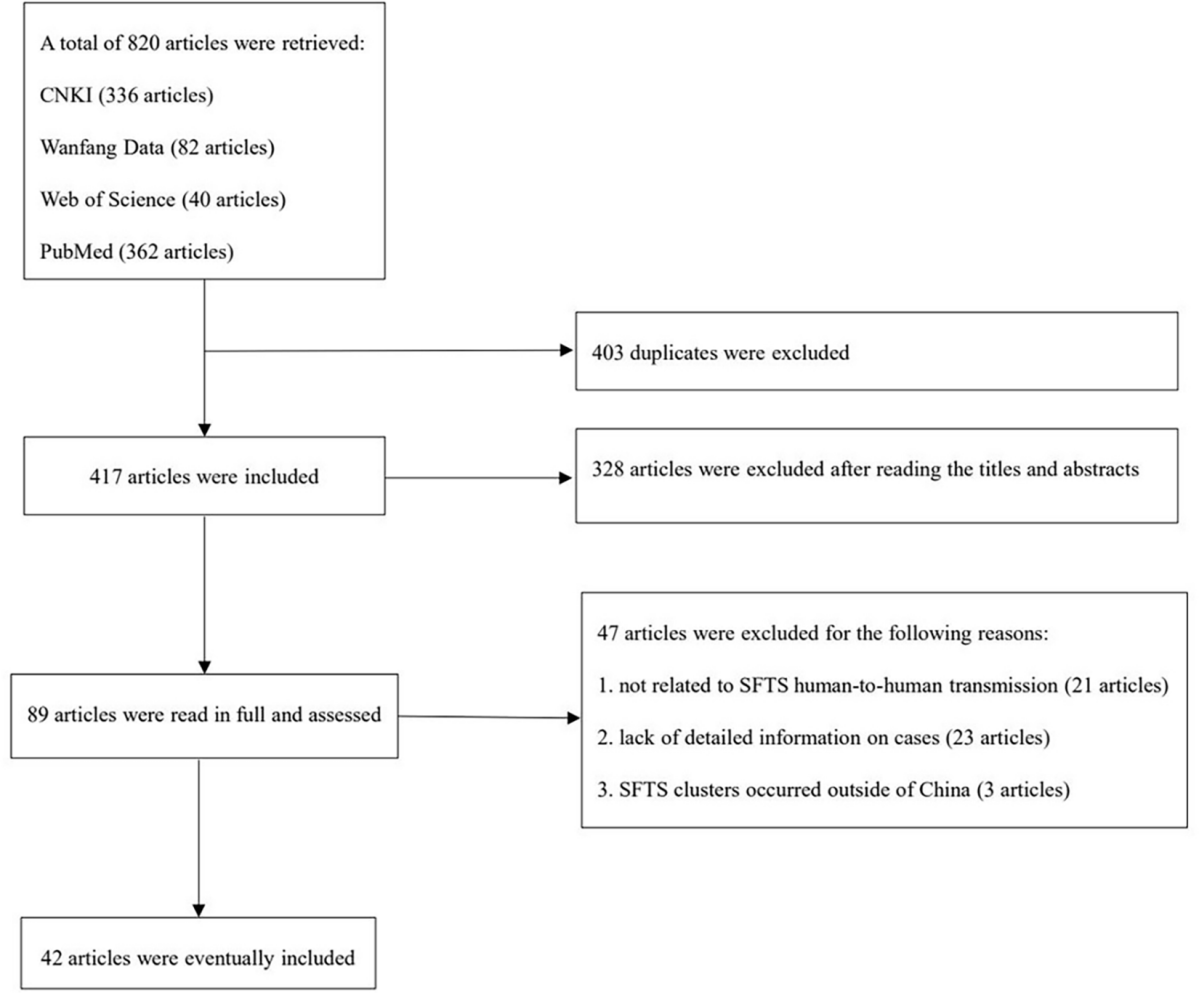
Literature screening flowchart.

Database searches yielded 820 potentially relevant articles. After removing duplications, 417 articles were included in the following screening. Of that, 328 were excluded after reading the title and abstract because of their apparent irrelevance. After reading the full text, 42 articles were finally included for further data extraction and analysis (Fig 1). The included articles were published from 2011 to 2024, 25 in Chinese and 17 in English.

### Overview of human-to-human transmission clusters

37 human-to-human transmission clusters were retrieved, which involved 172 cases, including 37 index cases and 135 secondary cases. The earliest cluster occurred in 1996 and the latest in 2023. Of 37 clusters, two clusters reported third-generation secondary cases, seven clusters involved 16 secondary asymptomatic infections (positive for nucleic acid and/or IgM antibodies to SFTS virus), and nine involved 17 healthcare workers. Of these, 35 clusters reported health outcomes of all involved cases, with an average case fatality rate of 31.33% (52/166). The number of cases involved in a single cluster ranged from 2 to 13, with an average of 5 cases.

### Regional distribution

The geographical distribution of 37 clusters showed regional clustering, mainly concentrated in rural areas in central, eastern and northeastern parts of China, involving 7 provinces. Of that, Jiangsu (9 clusters) accounting for the most clusters, accounted for 24.32%, followed by Henan, Shandong, and Zhejiang (6 clusters each), Anhui (5 clusters), Hubei (3 clusters), and Liaoning (2 clusters). In 2014, Zhejiang reported a cluster involving 13 cases and involved two provinces, Anhui and Zhejiang. In this cluster, two secondary cases in Anhui were diagnosed as confirmed SFTS cases after returning from a funeral for the index case in Zhejiang, and the source of the infection was suspected to be contact with the blood and secretions of the index case.

### Population distribution

Among the 172 cases involved in 37 clusters the youngest was 20 years old, the oldest was 86 years old, and the average was 56.85 years old. Among them, the age range of index cases was from 35 to 82 years old, with an average of 65.86 years old. Twenty cases were males and seventeen were females. The occupation was mainly farmers, accounting for 83.78% (31/37). The age of secondary cases ranged from 20 to 86 years old, with an average age of 53.45 years old. There were 82 males, 33 females, and 20 cases lacked gender information. The average age of index cases was older than that of secondary cases, and the difference was statistically significant (t = 4.992, *P* < 0.05) (Table 1).

**Table 1:** Comparison of basic information, disease course and death between the index and secondary cases of 37 clusters.

Characteristics	Index cases(n = 37)	Secondary cases(n = 135)	χ^2^	t	Z	P
sex, male	20(n = 37)	82(n = 115)	3.774	N/A	N/A	0.052
age, y	65.86 ± 10.78(n = 37)	53.45 ± 14.08(n = 103)	N/A	4.992	N/A	<0.05
age, ≥ 60y	27(n = 37)	42(n = 103)	11.289	N/A	N/A	<0.05
farmer	31(n = 37)	19(n = 51)	18.921	N/A	N/A	<0.05
fatal outcome	36(n = 37)	16(n = 129)	96.327	N/A	N/A	<0.05
disease duration, d	8.67 ± 2.41(n = 36)	12.68 ± 5.20(n = 54)	N/A	N/A	-4.131	<0.05

Note: Measurements are expressed as $\overset{―}{X}$±SD, y is year and d is day

χ^2^ is Pearson χ^2^ test, t is Independent Sample T test and Z is Wilcoxon W test

### Temporal distribution

**Fig 2:**
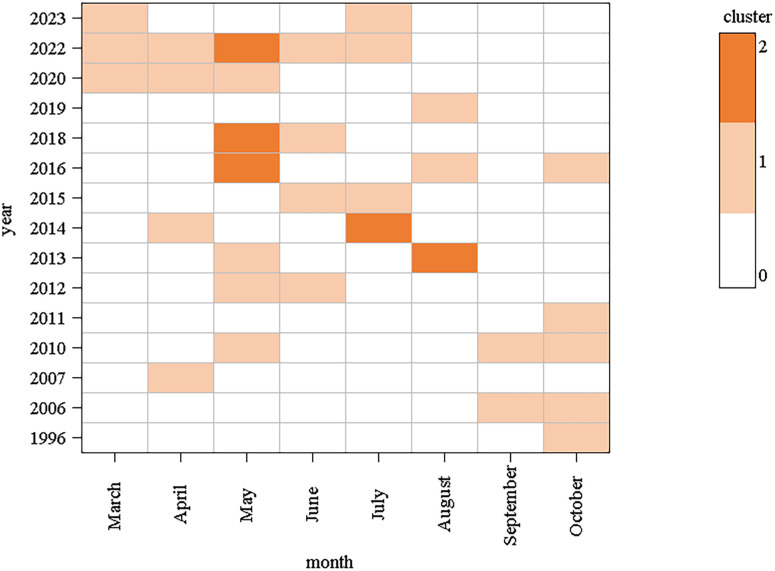
Temporal distribution of the index cases of the 37 clusters.

Based on the onset time of the index cases, 37 clusters occurred from March to October, with 3, 4, 10, 4, 5, 4, 2, and 5 clusters reported successively. Among them, the number of occurrences from May to July accounted for 51.35% (19/37). In terms of year of occurrence, the highest number of clusters were reported in 2022 (6 clusters), followed by 2016 (4 clusters) (Fig 2).

### Analysis of the onset and medical consultation of the index cases

The hospitals first consulted by the index cases were mainly primary medical institutions, of which 45.95% (17/37) were village-level clinics or health centers, 18.92% (7/37) were county-level hospitals, and 5.41% (2/37) were municipal-level hospitals. Multiple referrals to hospitals were common, with 78.38% (29/37) of cases having visiting frequency (or number of hospitals) ≥2 times, 79.31% (23/29) ≥3 times, and 13.04% (3/23) having 5 times. Of 37, 12 cases (32.43%) had a definite history of tick bites. Thirty-six of the thirty-seven index cases had symptoms recorded, which were mostly atypical, with fever (94.59%, 35/37), fatigue (51.35%, 19/37), and gastrointestinal symptoms (72.97%, 27/37) (Table 2), the latter being prevalent in diarrhea (8 cases), nausea (8 cases), and vomiting (8 cases). Leukopenia and thrombocytopenia were present in 100% (35/35) and 100% (33/33) of cases, with results of multiple tests in some cases suggesting the presence of progressive decrease with a minimum of 1.03×10^9^/ml in leukocytes and 6×10^9^/ml in platelets. 91.89% (34/37) mentioned bleeding manifestations. 81.48% (22/27) of cases were critically ill and died after giving up treatment and discharging from the hospital. 14.81% (4/27) of cases were discharged from the hospital after death. 3.70% (1/27) of cases were discharged from the hospital after recovery. 36 index cases died, except for 1 case that recovered, with a case fatality rate of 97.30%. The disease course of the 36 dead patients ranged from 4 to 15 days, with an average of 8 days.

**Table 2:** Basic information and clinical manifestations of index cases.

NO.	Reference NO.	Province	Onset date	Outcome	Tick bite	Fatigue	Fever	Gastrointestinal symptoms	Lymphopenia	Thrombocytopenia
1	[22]	Jiangsu	1996/10/2	death	unknow	yes	yes	yes	yes	yes
2	[6]	Anhui	2006/9/28	death	unknow	no	yes	no	yes	yes
3	[6]	Anhui	2006/10/31	death	yes	no	yes	yes	yes	yes
4	[23]	Jiangsu	2007/4/18	death	unknow	no	yes	yes	yes	yes
5	[24,25]	Henan	2010/5/20	death	no	yes	yes	yes	yes	yes
6	[26]	Shandong	2010/9/25	death	unknow	no	yes	yes	yes	yes
7	[23,27]	Jiangsu	2010/10/6	death	no	no	yes	yes	yes	yes
8	[28–31]	Shandong	2011/10/10	death	unknow	no	yes	no	yes	yes
9	[32]	Hubei	2012/5/6	death	unknow	no	yes	yes	yes	yes
10	[33]	Liaoning	2012/6/4	death	no	yes	yes	no	yes	yes
11	[34]	Zhejiang	2013/5/29	death	unknow	no	yes	yes	yes	yes
12	[35]	Shandong	2013/8/25	death	yes	no	yes	yes	yes	yes
13	[36]	Anhui	2013/8/27	death	yes	no	yes	no	unknow	unknow
14	[37,38]	Zhejiang	2014/4/23	death	yes	no	yes	no	yes	yes
15	[4]	Shandong	2014/7/6	death	no	yes	yes	yes	yes	yes
16	[5,39,40]	Liaoning	2014/7/28	death	unknow	yes	yes	yes	yes	yes
17	[41]	Shandong	2015/6/10	death	unknow	yes	yes	yes	yes	yes
18	[42]	Jiangsu	2015/7/10	death	unknow	yes	yes	yes	yes	yes
19	[43]	Jiangsu	2016/5/21	death	unknow	yes	yes	yes	yes	yes
20	[44]	Anhui	2016/8/9	death	yes	no	yes	yes	yes	yes
21	[45]	Jiangsu	2016/5/18	death	yes	yes	yes	no	yes	unknow
22	[45]	Jiangsu	2016/10/12	death	yes	yes	yes	no	yes	unknow
23	[46]	Henan	2018/5/7	death	no	yes	yes	yes	yes	yes
24	[47]	Zhejiang	2018/5/20	death	yes	no	yes	yes	yes	yes
25	[47,48]	Zhejiang	2018/6/12	death	unknow	yes	yes	yes	yes	yes
26	[49]	Jiangsu	2019/8/23	death	unknow	no	yes	yes	yes	yes
27	[50]	Anhui	2020/3/26	death	unknow	no	yes	yes	yes	yes
28	[51]	Jiangsu	2020/4/1	recovery	yes	no	no	no	unknow	unknow
29	[47]	Zhejiang	2020/5/9	death	unknow	yes	no	yes	yes	yes
30	[52,53]	Shandong	2020/6/12	death	yes	no	yes	yes	yes	yes
31	[54,55]	Henan	2022/3/12	death	unknow	yes	yes	no	yes	yes
32	[54,56]	Henan	2022/4/28	death	unknow	yes	yes	yes	yes	yes
33	[57]	Henan	2022/5/8	death	yes	yes	yes	yes	yes	yes
34	[57]	Henan	2022/5/20	death	unknow	yes	yes	no	yes	yes
35	[58]	Hubei	2022/7/16	death	yes	yes	yes	yes	yes	yes
36	[59]	Zhejiang	2023/3/24	death	unknow	no	yes	yes	yes	yes
37	[60]	Hubei	2023/7/12	death	unknow	yes	yes	yes	yes	yes

### Analysis of the secondary cases

The secondary cases in each cluster ranged from 1 to 12 cases, with an average of 4 cases. A total of 135 secondary cases were reported in 37 clusters, of which 35 clusters referred to the health outcome of the secondary cases, involving 129 cases, including 16 deaths, with an average case fatality rate of 12.40% (16/129). Most of the clusters occurred in the process of caring (72.97%, 27/37), mortuary (64.86%, 24/37), and rescuing patients (24.32%, 9/37), and the people involved could be relatives (75.76%, 100/132), medical workers (12.12%, 16/132), villagers (9.85%, 13/132) and morticians (2.27%, 3/132). Thirty-six clusters (97.30%, 36/37) were considered to be transmitted through contact with SFTS patient’s blood and bloody secretions, and one was presumed to be caused by contact with patient’s sweat, and three other clusters mentioned that some individual secondary cases had no direct contact with patient’s blood, and it was presumed that there might be the possibility of aerosol transmission. 12 out of the 37 clusters had deaths in secondary cases, of that, 10 clusters reported one death in each cluster, one cluster reported two deaths, and another had four deaths (Henan, 2022, 5 secondary cases). The case fatality rate of a single cluster ranged from 8.33% to 80%. One cluster in Jiangsu in 1996 and one in Shandong in 2014 reported third-generation human-to-human transmission. The shortest incubation period of the secondary cases was 5 days and the longest was 21 days, mostly within 2 weeks. The time between the onset of the last case and the onset of the index case in human-to-human transmission clusters ranged from 8 to 44 days, with an average of 18 days.

## Discussions

To better understand the occurrence process, epidemiological characteristics and clinical manifestations of SFTS human-to-human transmission clusters, this study systematically summarized and analyzed 37 clusters occurred in China which were publicly published between 2010 and 2023. Research findings showed that SFTS human-to-human transmission clusters have basically occurred in China every year since 2010, and the earliest one can be traced back to 1996, which suggests that SFTS virus infection has existed in the population for a long time. Most clusters involved second generation and very few involved third generation (2 clusters). Seven clusters reported 16 asymptomatic infections, among which all the third-generation infections in one cluster were asymptomatic.

In this study, we found that clusters were mainly distributed in Jiangsu, Henan, Shandong, Zhejiang, Anhui, Hubei, and Liaoning provinces, and the above 7 provinces are also the main endemic areas of SFTS in China, where about 99% of the cases occurred [8]. It should be noted that, in addition to the above high prevalence areas, other provinces or regions with relatively low numbers of reported cases have also reported family clusters of SFTS [61]. Although it is not clear whether the disease is caused by co-exposure or human-to-human transmission, it is necessary to pay more attention to the possible human-to-human transmission. Among 37 clusters, the index cases were mostly infected in rural areas, and most of them were farmers (83.78%). In mountainous areas, hilly regions and other areas with abundant vegetation, the density of ticks is high. Farmers often have to work and go in and out of these areas, so they are at a higher risk of exposure compared to other groups More than half of human-to-human transmission clusters in this study occurred from May to July. At this time, the temperature rises, and the activity of ticks enters a peak period. The opportunities for people to come into contact with ticks increase, and the risk of infection rises significantly [62,63]. In this study, the earliest index case occurred in March and the latest in October, and the cluster caused by the latter occurred in November, suggesting that close attention still needs to be paid to the occurrence of human-to-human transmission clusters during the non-major endemic seasons of SFTS.

It was found that the incubation period of the secondary cases of clusters ranged from 5 to 21 days, mostly within 2 weeks, which was basically consistent with the incubation period of infections transmitted by tick bite (generally 1–2 weeks, with an average of 9 days) [64]. Early symptoms of the index cases are mainly fever, fatigue and gastrointestinal symptoms, which were easy to be regarded as common diseases such as cold or diarrhea, and then take medication by themselves or go to primary medical institutions such as village health clinics and private clinics for medical treatment. Due to the atypical early symptoms and the limitations of primary medical institutions in identifying this disease, there are cases of misdiagnosis and underdiagnosis [65]. The study found that most patients seek medical treatment in many places as their conditions don’t improve or even worsen. More than 79% of the cases had visited hospitals three times or more, which increased the risk of human-to-human transmission due to the delay in case diagnosis. This finding was similar to that in South Korea [66]. Some index cases were identified as SFTS cases only when the disease occurred among the contacts [6,22–29]. Early identification by primary healthcare institutions is of great significance for the prevention and control of SFTS. It is recommended that primary medical institutions, especially those in key epidemic areas, should give health tips and carry out routine blood tests for the disease when suspected fever cases appear in daily consultations, so as to achieve early diagnosis, treatment and management, reduce the case fatality rate, and avoid clusters.

Among 37 index cases, except for one case of recovery, 36 died of SFTS, with an extremely high case fatality rate (97.30%). In addition, the case fatality rate monitored in secondary cases (12.40%) was also much higher than the average case fatality rate reported nationally (5%) [67]. The high case fatality rates of clusters, especially the index cases, are considered to be related to the old age of the cases, the lack of timely diagnosis and treatment, and the high viral load [68], which is also one of the main factors contributing to human-to-human transmission. In this study, almost all of the index cases had different degrees of hemorrhage, and the secondary cases mostly occurred among relatives, medical workers, villagers and morticians who were involved in nursing care (72.97%), funeral and mortuary (64.86%), and medical treatment (24.32%). Infections were mainly caused by contact with SFTS patient’s blood and bloody secretions (97.30%), while a few of the secondary cases claimed that they had not contacted the patient’s blood and bloody secretion, suggesting the possibility of aerosol transmission in confined spaces [38]. A cluster of SFTS human-to-human transmission that occurred in South Korea also confirmed this possibility [69]. The findings suggest that is necessary to further strengthen health promotion and education, raise public awareness of SFTS, improve personal protection awareness when caring for SFTS patients, reduce funeral procedures and advocate safe funerals. Nine clusters involved secondary infections among medical workers, which implies that it is necessary to further enhance diagnostic awareness and capacity, strengthen personal protection training for medical workers and avoid nosocomial infections [70].

There are some limitations in this paper. Firstly, during the process of literature screening, literature that lacked details and that could not yet be distinguished whether the cluster was caused through human-to-human transmission [71,72] were excluded. For example, in one cluster there were two patients who were couple lived together in rural areas and had developed SFTS successively. Epidemiological investigations had shown that there was a high possibility that they both was transmitted through tick bites, but it could not be ruled out whether it was transmitted through close contact because they had close contact during the onset of disease [72]. Secondly, the data in this study were taken from published literature, and unpublished clusters of human-to-human transmission, such as the cluster in Anhui that involved 18 cases [73] and the cluster that occurred in Hunan and other places were not included, which to some extent underestimated the actual level of SFTS human-to-human transmission.

In summary, this study extracted data on SFTS human-to-human transmission by screening publications and analyzed the epidemiological characteristics, occurrence process and risk of transmission, in order to provide a basic knowledge for the scientific prevention and control of SFTS human-to-human transmission.

## Supporting information

S1 Dataset Data used in this study. (XLSX)
